# Bending Limit Tests for Ultra-Thin Liquid Crystal Polymer Substrate Based on Flexible Microwave Components

**DOI:** 10.3390/mi9100531

**Published:** 2018-10-20

**Authors:** Yu Lan, Yuehang Xu

**Affiliations:** School of Electronic Science and Engineering (National Exemplary School of Microelectronics), University of Electronic Science and Technology of China, Chengdu 611731, China; Lanyu136482942@163.com

**Keywords:** bending limit test, flexible filters, microwave components, LCP substrate, bending effect

## Abstract

In this paper, bending limit tests for one ultra-thin liquid crystal polymer (LCP) substrate (Rogers 3850) based on the mechanical properties of flexible microwave microstrip components are presented. First, a set of 50 Ω microstrip lines, a band-pass filter, and a stepped impedance filter in X-band, are designed by using double clapped LCPs with 50 μm thickness of substrate and 18 μm thickness of copper, which is fabricated by conventional photolithography. Then, the limit tests of the flexibility of the LCP microwave microstrip components are presented, and the range of the bending limit radius, from 1 mm to 0.75 mm, is demonstrated from the testing results. It is found that the cause for component failure is fracture of the copper (18 μm thickness) laminate, according to the bending limit test experiments. Finally, the analysis of the reasons for the collapse of the microwave components, under bending situations, is explored. The results from this work would be useful for further designs of the flexible microwave devices and systems on LCP substrates, with compact sizes and good performance.

## 1. Introduction

As the development of flexible electronic devices and system modules, widely concerned in radio frequency (RF)/microwave/millimeter-wave and biomedical applications, are increasing, flexible materials have been attracting wide attention in high frequency applications. Liquid crystal polymer (LCP) substrate has also been showing its superiorities in some applications of microwave and millimeter-wave circuits, due to its features of excellent loss tangent (approximately 0.005 over the entire RF range up to 170 GHz) [1,2], stable and desirable electrical characteristics for wide band, extremely low moisture absorption, and low thermal expansion coefficient. The ultra-thin substrate and good dimensional stability of LCP make it show much excellent bending and stretching characteristics than other traditional rigid dielectric substrates. Ease of bending also makes this material suitable for reel-to-reel processing and conformal applications, applicable for non-planar surroundings. In the past decade, many millimeter-wave components, emerging structures, and system modules based on LCP substrates have been reported [3,4,5,6,7,8]. These applications show that LCP substrates could cover the frequency range from 1 GHz to 100 GHz, and the circuits on LCP substrates always had the advantages of light weight, low-cost packaging, and easy fabrication.

Recently, some applications of LCPs, regarding bending tests, had been presented, such as a compact broadband flexible antenna utilized in wireless local area network (WLAN) and upper ultra-wideband (UWB) systems fabricated on LCPs at a thickness of 127 µm [9]. A bent transmission line and a 20 GHz interdigital coupled lines filter on the LCPs [10] were demonstrated in bending and flat situations. Inkjet printing technology was introduced for the fabrication of flexible circuits with the advantages of more convenience, low-cost, and much more stability, a K-band hairpin band-pass filter [11], a 25 GHz narrow-band band-pass filter [12], and a 26 GHz–33 GHz series-fed two-dipole antenna comprising a balun filter as the feeding port [13], based on an LCP substrate with thickness of 100 µm that had been presented using inkjet printing technology. In our previous works [14,15], an interdigital two-stage hairpin band-pass filter, based on LCPs which consist of two half-wavelength (λ/2) microstrip line resonators with intercoupling method, was introduced, with compact size and good performance, and the bending effect research shows that the S-parameters of the filter are not sensitive to bending behavior.

All the above references addressed the outstanding flexibility of LCP substrates, but they did not conduct limit bending tests to characterize a limit tolerance of microwave devices on LCP substrates. The limit bending tolerance would be a very significant parameter for future flexible applications under repeated bending and stretching conditions, such as wearable devices mounted on the skin, collecting the health information from bodies [16,17], and high-speed wireless communication systems integrated in clothes or other folding objects, in the future.

This paper goes further on the development of the bending tests presented in [14,15], and demonstrates further results from bending limit tests for this LCP substrate. Indeed, for getting the bending limit parameters before the circuits collapsed, the breakdown tests were used to extract the bending limit radius. Finally, in order to pave the way for the further design of the flexible circuits, the failure mechanism of these components are researched and presented, giving the further design process some guidelines to avoiding time-consuming and cost-consuming experiments.

This paper is organized as follows: Section 2 presents the design processes and experimental results of 50 Ω microstrip lines, band-pass filters, and low-pass filters. Section 3 presents the bending limit test experiments and discussion of the reasons for the interface failure and sharply deteriorating performance. Finally, the conclusion for this work is presented in Section 4.

## 2. Device Design

**Figure 1:**
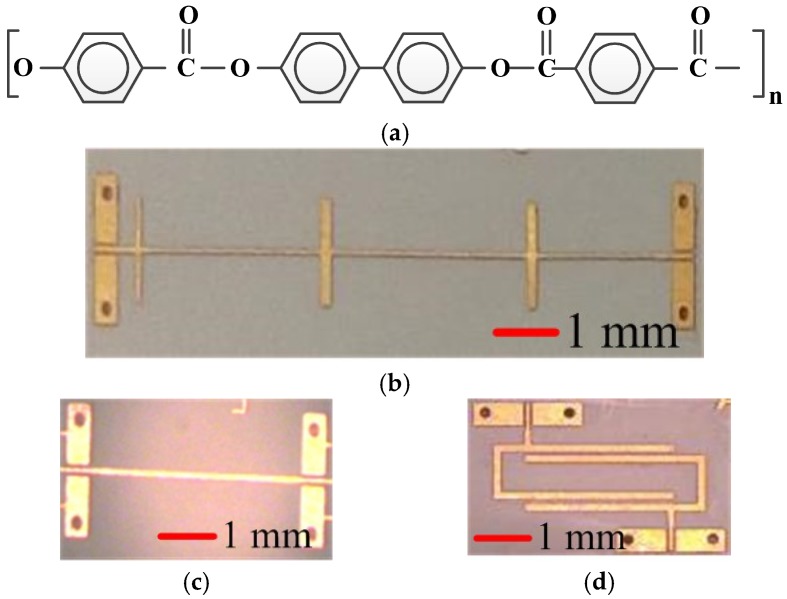
(**a**) Chemical structures of liquid crystalline polymer, (**b**) photograph of the 10GHz stepped impedance low-pass filter, (**c**) image of the 50 Ω microstrip line, and (**d**) the 9.5 GHz band-pass filter.

Transmission lines are always the critical components for microwave and mm–meter wave circuits, which are mandatorily used for building almost all passive circuits, including matching networks, hybrid couplers, baluns, filters, etc., so high-performance and more compact transmission lines in different technologies have the same aim of reducing the area of the devices and getting a higher integration level. In this application, the LCP substrate 3850 [18] is offered as a double copper-clad laminate with constant relative permittivity of 3.0, stable loss tangent of 0.0025, substrate thickness of 50 µm, and copper laminate thickness of 18 µm. The chemical structure of the LCP substrates are shown in Figure 1a. The width of 50 Ω microstrip line is 120 µm, as shown in Figure 1c. A 10 GHz stepped impedance low-pass filter consists of six different length microstrip lines with high impedance of 60 Ω (microstrip width 0.08 mm) and low impedance of 5 Ω (microstrip width 2 mm), as shown in Figure 1b. The high impedance ratio between high impedance microstrip line and low impedance line always offer high performance for low-pass filter, but the width of 0.08 mm had reached our PCB fabrication limit. A band-pass filter consists of two identical half-wavelength (λ/2) microstrip lines, and two tapped I/O lines at each side of λ/2 microstrip lines, as shown in Figure 1d. This kind of symmetric feed structure is commonly used [19] to generate two transmission zeros in the stop-band. The ground coplanar waveguide (GCPW), with a 0.085 mm-wide center strip and a 0.075 mm-wide gap, was employed as the input and output of the RF signals. The reasons of selecting a 50 Ω microstrip lines, stepped impedance low-pass filters, and interdigital band-pass filter as the geometry of bending test, is that the microstrip lines and filters are the foundation of microwave PCB circuits. Meanwhile, we can also study how the bending behavior affects the coupling effects of the microwave circuits, by comparing the stepped impedance and interdigital filters.

In the measurement process, the characteristics of the microstrip lines and microwave filters were characterized through ground–signal–ground (GSG) probes (Cascade 250 µm pitch) connected to an HP E8364C vector network analyzer (VNA, Agilent, Palo Alto, California, USA) on a probe station. More details about the engineering design processes are presented in our previous publications [14,15].

## 3. Bending Limit Tests and Discussion

**Figure 2:**
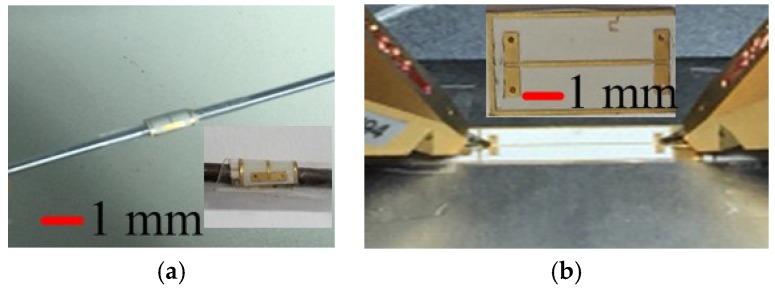
Flow of measurement setup of microstrip line. (**a**) Warp it onto the surface of the cylinder and (**b**) open and stick it on a plate metal on the probe station.

In Section 2, the introduced bending vehicles are semi-cylinders, and the filters were fixed onto the surface of the semi-cylinder to achieve bending effects. However, when the radius of the semi-cylinder reduces to a certain degree, the filters cannot be fixed to the bending vehicles anymore. To testing the bending limit radius, a cylinder vehicle with radius of 1.5 mm, 1 mm, and 0.75 mm was used. The radius of the cylinder is much smaller than the previous semi-cylinder vehicle, with the minimum radius of 5 mm. The microstrip lines and filters were wrapped onto the surface of the cylinder using strong adhesive transparent tapes, then opened and glued on the probe station to testing the S-parameters. Figure 2 shows the bending test flow of the 50 Ω microstrip lines.

### 3.1. Results of Bending Limit Tests of the Microwave Components

**Figure 3:**
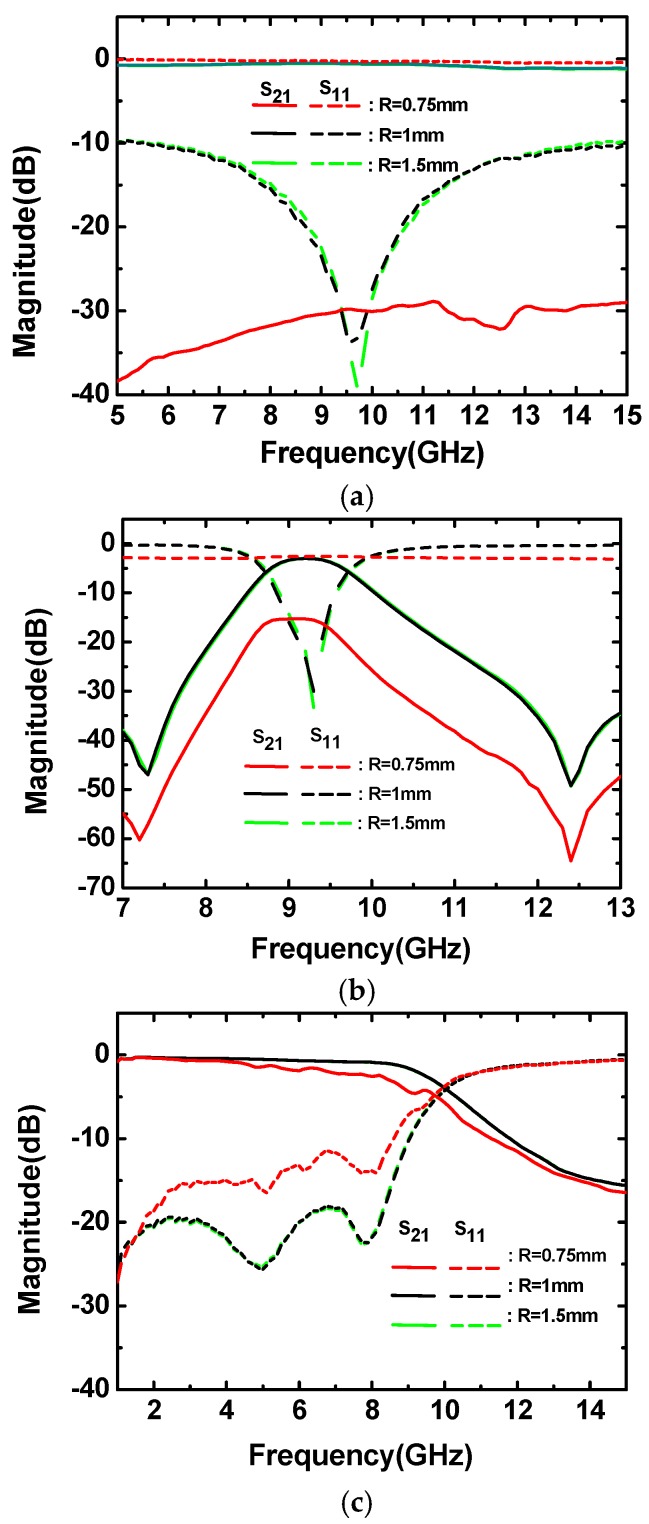
The limit test results (radii of 1.5 mm, 1 mm, and 0.75 mm). (**a**) Results of the 50 Ω microstrip lines. (**b**) Results of the band-pass filters. (**c**) Results of the low-pass filters.

Following the test flows, Figure 3 shows the bending limit test results versus frequency for the 50 Ω microstrip lines, band-pass filters, and low-pass filters. In Figure 3, it can be seen that the performance of the microstrip lines and filters has little deterioration under the bending radii from 1.5 mm to 1 mm. However, when the radius was reduced to 0.75 mm, the insertion loss of microstrip lines was sharply increased to 30 dB, showing complete reflection phenomenon; the insertion loss and return loss of the band-pass filters deteriorated to 15 dB and 3 dB in the pass-band, respectively; the maximum insertion loss of the low-pass filters also deteriorated from 1.5 dB to 4.5 dB at 9 GHz. The deteriorated performance of all the components was so severe that they were no longer able to work normally.

Hence, the range of radius, from 1 mm to 0.75 mm, can be estimated as the limit bending radius for this LCP substrate, meaning that any kind of bending behavior with bending radius less than 1 mm might disable components, devices, and systems based on this LCP substrate.

### 3.2. Study and Discussion of the Failure Mechanism

**Figure 4:**
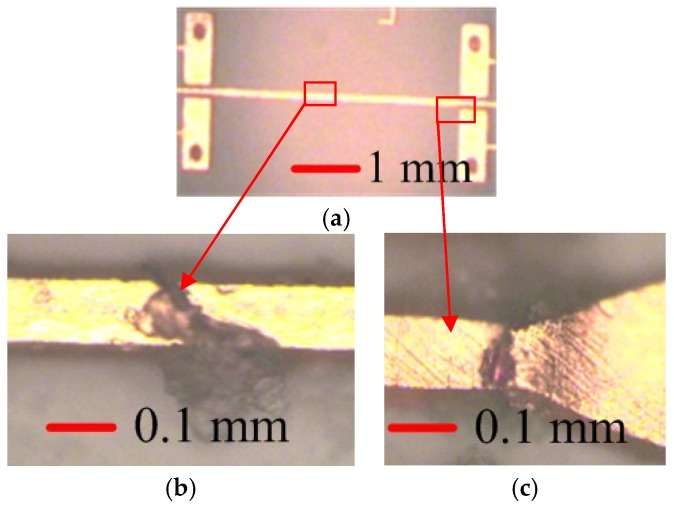
Microscope images of the cracked points. (**a**) Zoomed out image after the bending test, where the squares are the breaking points; (**b**) cracked point along the microstrip line; and (**c**) cracked point at the transition of microstrip line and ground coplanar waveguide (GCPW).

In the bending limit tests for the microwave components, the frequency shift and increased insertion loss are observed, which is caused by a mechanical deformation of the metallization microstrip. When the microstrip line is under bending situation, the copper trace of the microstrip lines would be stretched, and the thickness and the roughness would become thinner and non-uniform, respectively. The stability of the metallization to LCP substrate adhesion would also be reduced, leading to a higher sheet resistance [17]. When the stretched force approached its critical point, the microstrip lines are broken, and the microwave components’ performance collapses. To research the failure mechanism of these components, the breakage of metallization microstrips after bending tests are observed under the microscope. Figure 4 shows the images of cracked points along the microstrip line and transition junction between the microstrip line and ground coplanar waveguide (GCPW) feeding ports.

**Figure 5:**
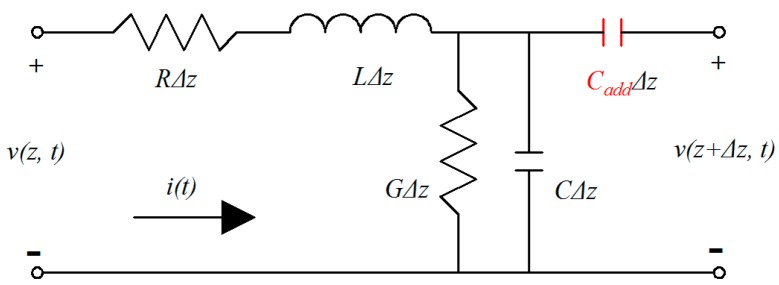
The lumped equivalent circuit of the collapsed transmission lines [21].

From Figure 4, it can be seen that the microstrip lines cracked along the signal trace, which is equal to introducing a capacitance (Cadd) along the signal trace [20], as shown in Figure 5. The gaps suddenly change the impedance of the reference plane at the cracked points, which is the reason for the insertion loss sharply increasing, and the complete reflection phenomenon of the microstrip lines and the band-pass filters.

As we can see from the results in Figure 3 and Figure 4, under high strain level, the straight microstrip line is fragile, and the coupling structure is much more sensitive than the direct connecting structures. In future flexible microwave circuits and systems, the mender microstrip lines can offer more robust performance under high strain levels, and the coupling structures may not be suitable for high strain level applications.

For the interface failure between the flexible substrates and films, three types of failure modes, which are fracture, slipping, and delamination, are presented [21], depending on different materials and various combinations of the thickness of flexible substrates and films. It can be expected that the thickness of films would play a very important role for the types of failure modes under bending situations. Cracking mode would occur when the films are very thin relative to the flexible substrate’s thickness; then, as the films’ thickness increases, the slipping mode appears; if increasing the films further, whole films would be exfoliated from the substrate, forming the delamination mode.

In this case, the copper thickness is 18 μm, and the substrate thickness is 50 μm for the LCP substrate 3850 (Rogers Corporation, city, state, USA). It is possible that the slipping or delamination failure modes should be observed according to the theory in [21], but the only failure mode of fracture was observed. The reason for that is that the strength of the adhesive bonding between films and substrates is another sensitive parameter for the types of failure modes. The LCP 3850 is a commercialized version from Rogers Corporation, so the strength of the adhesive bonding is excellent, avoiding the slipping and delamination between the film and the substrate.

It can be seen from the Figure 3 that the performance of the microwave components has little deterioration, even under the bending radii of 1 mm. However, when the radius decreased to 0.75 mm, the devices collapsed suddenly without appreciable signs of transition. The reason is that the flexible components have bare deterioration, even under the bending radius of 1 mm; however, the components sharply collapsed when the bending radius was reduced to 0.75 mm.

## 4. Conclusions

In this paper, bending limit tests were carried out for LCP substrate, and the results show that the bending limit radius range is about from 1 mm to 0.75 mm for this LCP. Then, the failure mechanism and reasons for the collapsing of these microwave circuits are also studied. This paper revealed the bending limit parameters for LCP substrate in microwave range, which would be a significant assessment for future high-frequency flexible devices and systems based on an LCP substrate.
